# Isoflurane Is Effective for Anesthesia of the Greater Wax Moth (*Galleria mellonella*) Larvae

**DOI:** 10.3390/insects17060600

**Published:** 2026-06-08

**Authors:** Jakub M. Kwiecinski

**Affiliations:** Department of Microbiology, Faculty of Biochemistry, Biophysics and Biotechnology, Jagiellonian University, 30-387 Krakow, Poland; jakub1.kwiecinski@uj.edu.pl

**Keywords:** *Galleria mellonella*, wax moth, isoflurane, insect, anesthesia, euthanasia

## Abstract

*Galleria mellonella* (wax moth) larvae are increasingly used as model organisms in biomedical research, yet ethical guidelines for their anesthesia and euthanasia are lacking. A review of published papers revealed that most projects involving *G. mellonella* either did not use any anesthesia or euthanasia, or employed suboptimal methods that may cause pain. This study demonstrates that brief exposure to isoflurane at 2 μL/cm^3^ provides rapid, long-lasting anesthesia without impairing immune functions of the larvae. Additionally, combining isoflurane anesthesia with subsequent freezing at −20 °C offers an effective euthanasia method. The described anesthesia and euthanasia methods are simple, scalable, humane, and well-suited for laboratory use in *G. mellonella*, supporting improved welfare standards in invertebrate research.

## Simple Summary

Growing evidence that invertebrates are capable of experiencing pain has led to increased concern for the welfare of insects that are farmed for food, kept as pets or zoo animals, or used in biological research. However, there is still a lack of clear guidelines and techniques for preventing insect pain. This study describes an effective, humane, and simple method for anesthesia and euthanasia of greater wax moth (*Galleria mellonella*) larvae, one of the most widely used insects in microbiology and toxicology research.

## 1. Introduction

Larvae of the greater wax moth (*Galleria mellonella*) have become a popular invertebrate model for research in microbiology, toxicology, and insect biology [1,2]. This widespread use of *G. melonella* as an alternative laboratory animal raises new ethical challenges. Growing evidence that invertebrates are capable of experiencing pain highlights the importance of considering insect welfare [3,4,5,6]. Unfortunately, the biomedical research community is still slow to develop appropriate procedures and guidelines ensuring the welfare of laboratory insects [7,8]. Indeed, guidelines for anesthesia and euthanasia in *G. mellonella* are lacking, despite the use of potentially painful procedures, and the need to dispose of live larvae after experiments.

Data on insect anesthesia and euthanasia remain limited. While chilling and freezing are commonly used (probably because they seem to provide visible animal immobilization), low temperature alone does not provide analgesia [7,9]. Exposure to cold might actually even induce pain in some invertebrates [6], possibly further potentiated by ice crystal formation inside tissues. Therefore, organizations like the American Veterinary Medical Association and Insect Welfare Research Society do not consider freezing to be humane, and discourage its use as a sole euthanasia method for invertebrates [10,11]. A few studies have examined different methods of insect anesthesia (e.g., carbon dioxide, isoflurane) and euthanasia (e.g., injection of pentobarbital, potassium chloride, or ivermectin; immersion in soapy water, ethanol, or formalin; freezing; inhalant anesthetic overdose) [12,13,14,15,16,17]. Results often varied across insect taxa, even between closely related species [6,12,17], emphasizing the need for species-specific guidelines.

An ideal method for *G. mellonella* should ensure rapid loss of consciousness, allow control of anesthesia duration, be inexpensive, require no specialized equipment, allow simultaneous treatment of multiple animals, be reversible, and minimally impact the studied physiology. This article identifies inhalant anesthesia with isoflurane at 2 μL/cm^3^ and a two-step euthanasia process (overnight incubation of anesthetized larvae at −20 °C) as optimal approaches meeting these criteria.

## 2. Materials and Methods

### 2.1. Literature Review

To identify anesthesia and euthanasia methods currently used in *G. mellonella* research, the PubMed database was searched with the “*Galleria mellonella*” query. After limiting results by year to 2024 only, 376 English-language articles were identified, and 359 were successfully accessed and included in the analysis. Of these, 347 reported actual experimental work on *G. melonella*.

### 2.2. Animals

Final instar *G. mellonella* larvae were purchased from a local pet food supplier (where they were reared on honeycombs, without antibiotics), brought to the laboratory in a plastic transport container filled with wood shavings with an ultra-fine steel wire cap for ventilation, and kept in darkness at 30 °C in the same container for 24–48 h before experiments to acclimatize to laboratory conditions. Experiments were performed in a minimum of two independent repeats, at 30 °C, on larvae weighing 150–300 mg, with individuals of similar weight evenly distributed between experimental groups.

According to Polish law, no ethics approval is required for *G. mellonella* experimentation. The project falls within the principle of 3Rs (Replacement, Reduction, Refinement), as its goal is to refine animal experimentation by minimizing the distress of *G. mellonella* during experimental procedures.

### 2.3. Anesthesia and Euthanasia

**Figure 1:**
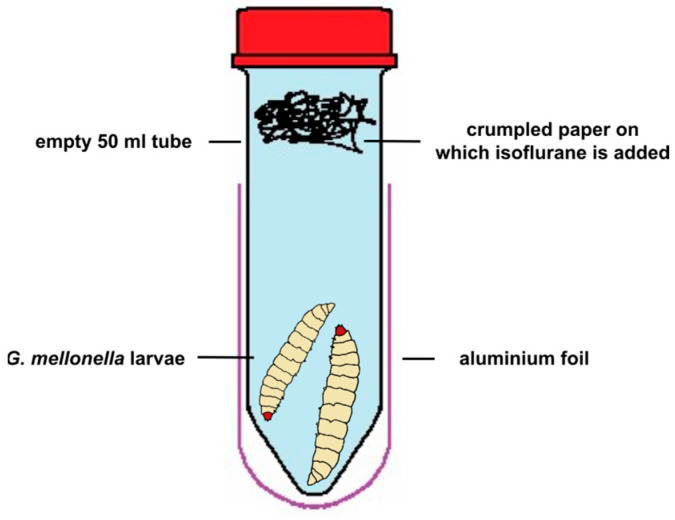
A simple setup was used to test the effects of isoflurane on *G. mellonella* larvae.

A 50 cm^3^ (50 mL) plastic tube served as the anesthesia chamber (Figure 1). Three to four larvae were placed at the bottom, and the lower part of the tube was wrapped in aluminum foil to provide comfortable darkness for larvae. A crumpled piece (~70 cm^2^) of two-ply paper towel was inserted into the tube opening. Indicated volumes of liquid isoflurane (VetPharma, Barcelona, Spain) were applied directly onto the paper, and the tube was tightly capped.

For batch experiments, time to induction of anesthesia (the point when all larvae in the tube ceased moving and reacting to tube shaking) was recorded. Larvae were either removed from the tube into ambient air immediately after induction or left inside for a specified additional time. Recovery time (to reappearance of spontaneous movement) was recorded for each larva. For experiments where larvae were treated individually, the same setup was followed, but the time to onset of anesthesia was defined as a point when larvae ceased to move and react to tube shaking and prodding with a blunt needle, while the time to recovery was defined as the time to the reappearance of any response to prodding with a blunt needle.

For euthanasia experiments, larvae were monitored for 2 h after removal from the chamber, and additionally at 24 h after removal. Larvae were considered dead if no spontaneous movement or response to touch was observed at any of these times. In some experiments, the entire tube containing anesthetized larvae was transferred to −20 °C overnight immediately after induction of anesthesia.

### 2.4. Evaluation of Immune Functions

To measure immune responses, hemolymph was collected and pooled from 3 to 4 conscious or anesthetized animals into pre-chilled tubes by piercing a foreleg with a needle. Samples were diluted 8-fold with ice-cold insect anticoagulant physiological saline (150 mM NaCl, 5 mM KCl, 10 mM Tris-HCl, 10 mM EDTA, 30 mM sodium citrate, 10 mM glucose, pH = 6.9). Viable hemocytes were counted in a Bürker chamber with trypan blue. To measure melanization, 50 μL of diluted hemolymph was mixed with 50 μL of phosphate-buffered saline containing 2.5 × 10^5^ CFU of *Candida albicans* ATTC 10231 in a 96-well plate, incubated at 37 °C for 1 h, and melanin production was measured as absorbance at 405 nm [18]. To measure the survival of microbial infections, larvae were injected with a Hamilton-type syringe through their last proleg with 10 µL of inoculum containing 5 × 10^5^ CFU of either *C. albicans* ATTC 10231, or *Staphylococcus aureus* LAC in saline. Larvae were afterwards placed in plastic Petri dishes in darkness at 30 °C, and their mortality was measured once per day for up to 3 days after injection. No mortality was observed in control larvae injected with pure saline.

### 2.5. Statistics

All data were pooled from a minimum of two independent experiments. Differences between groups were analyzed by one-way ANOVA (with either a posttest for linear trend, or a Dunnett’s multiple comparison posttest), by an unpaired *t* test, or by the Mantel–Cox logrank test. Two-tailed *p* values were used, and Prism 7 (Graph Pad Software, Boston, MA, USA) was used for calculations.

## 3. Results

### 3.1. Anesthesia and Euthanasia in G. mellonella Literature

Analysis of 347 research articles published in 2024 that used *G. mellonella* confirmed its expanding role as an alternative animal model. Most studies focused on microbiology (68%—of these, regarding studied microorganisms, 83% investigated bacteria and 18% fungi; regarding studied topics, 59% examined virulence mechanisms and 48% antimicrobial agents), followed by toxicology (22%) and insect biology (11%, including studies of anti-insect compounds and the biology of entomopathogenic nematodes). Another 2% addressed immunology, anti-inflammatory and antioxidant drug development, and biomedical imaging (note that as some articles covered multiple topics, the total added percentage exceeds 100%).

The vast majority of studies (89%) did not mention any form of anesthesia or humane killing, despite involving potentially painful procedures (hemolymph extraction, dissection, slow grinding, and—in single instances—burning and superficial abrasion), and typically ended with live larvae requiring disposal after experiments. This reflects the broader issue: despite growing interest in invertebrate ethics, biomedical researchers remain less likely than invertebrate veterinarians to consider insect welfare, and reporting standards on invertebrate welfare during experiments are lacking [7,8]. Notably, 8% of studies homogenized some of the larvae as part of the study (mostly in order to extract nucleic acids, proteins, or microorganisms for further analysis). Instantaneous mechanical homogenization has been proposed as a humane method for the slaughter of insects reared for food [19,20], but it was often unclear how instantaneous the homogenization death was in the analyzed studies, and it was usually preceded by submerging larvae in various homogenization buffers, possibly inducing additional stress. Most importantly, homogenization was never used intentionally as a means of euthanasia, but rather as part of experimental protocols meant to extract the larvae’s contents.

Only 7% of articles reported attempts at anesthesia before painful procedures, and just 5% described intentional euthanasia at study completion. Almost all of these relied solely on cooling or freezing—methods that immobilize insects but probably do not eliminate pain, which leads veterinarians to discourage their use without prior anesthesia or analgesia [7,9,10]. Only three studies used other approaches: decapitation, ethanol, and—most interestingly—isoflurane anesthesia [21]. These findings prompted the present study to evaluate and optimize isoflurane use in *G. mellonella*.

### 3.2. Isoflurane for Anesthesia

**Figure 2:**
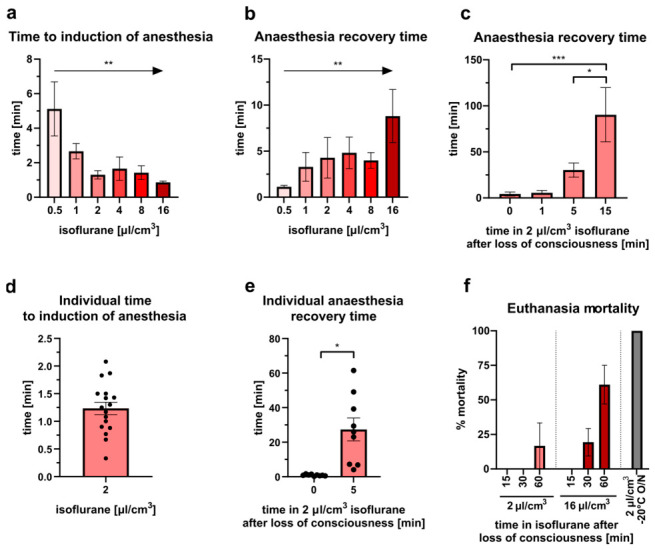
(**a**) Anesthesia induction and (**b**) recovery times were measured at different isoflurane doses on *G. mellonella* larvae in batches. The impact of prolonged isoflurane exposure on (**c**) anesthesia duration and (**f**) mortality, with optional added overnight incubation at −20 °C, was also tested. Additionally, (**d**) time for anesthesia induction and (**e**) recovery was measured for larvae that were anesthetized individually rather than in batches. All results come from a minimum of two pooled independent experiments; n = 3 (**a**,**f**), n = 6–9 (**c**), n = 9 (**b**,**e**); n = 17 (**d**); differences between groups were analyzed by ANOVA with a posttest for linear trend (**a**,**b**), by ANOVA with a Dunnett’s multiple comparison posttest (**c**), or by an unpaired *t* test (**e**); * *p* < 0.05; ** *p* < 0.01; *** *p* < 0.001; data are shown as mean ± SEM; optionally, individual values are also displayed (**d**,**e**).

Isoflurane proved highly effective as an anesthetic for *G. mellonella* larvae. At the lowest tested dose of 0.5 μL/cm^3^, anesthesia was induced in about 5 min, with induction time progressively decreasing to just over 1 min at 2 μL/cm^3^ (Figure 2a). As this dose exceeds the theoretical saturation of isoflurane vapor (~1.6 μL/cm^3^) [15], a further dose increase had a minimal effect on induction time. No visible signs of distress (tremors, hyperexcitation, regurgitation) were observed during exposure and anesthesia induction, and larvae remained anesthetized as long as they remained in the chamber.

Recovery time after removal from the chamber was dose-dependent (Figure 2b). Some animals in the 8 and 16 μL/cm^3^ groups regurgitated during recovery, but no distress signs were observed in groups with lower isoflurane doses. Recovery time was significantly prolonged when larvae remained in the chamber after the onset of anesthesia (Figure 2c).

The experiments described above relied on the scenario where several larvae were placed together in the tube; therefore, the recorded “time to induce anesthesia” reflected the time needed to anesthetize the most resistant of the animals in the batch. Nevertheless, when the anesthesia of individual larvae was tested in a separate experiment, the average time to induction of anesthesia at the saturating isoflurane dose of 2 μL/cm^3^ was quite similar to that recorded for the batch treatment: slightly above 1 min, with variation ranging from under half a minute to over two minutes (Figure 2d). When the recovery time of animals anesthetized in this individual way was measured, the results once again resembled those obtained with batch treatment. Recovery of larvae removed from the chamber immediately after the onset of anesthesia was almost instantaneous (approximately 1 min), while larvae left in isoflurane for an additional 5 min after the onset of anesthesia needed on average 30 min to recover, with times ranging from 5 min to over an hour (Figure 2e).

### 3.3. Isoflurane Plus Freezing for Euthanasia

Isoflurane alone was insufficient for reliably euthanizing *G. mellonella* larvae, even at 16 μL/cm^3^ and 60 min exposure (Figure 2f). In contrast, a two-step euthanasia protocol (induction with 2 μL/cm^3^ isoflurane followed by overnight freezing at −20 °C) was consistently effective, with 100% mortality.

### 3.4. Isoflurane Anesthesia and Immune Functions

**Figure 3:**
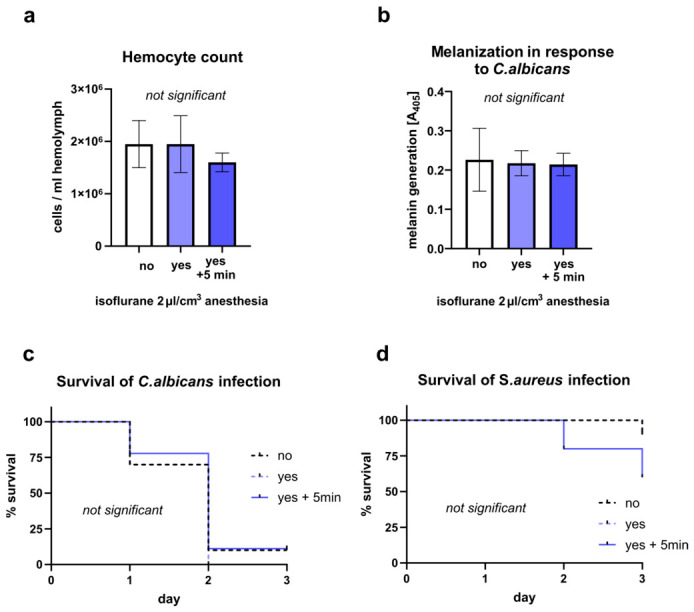
Immune functions were evaluated through (**a**) hemocyte counts, (**b**) hemolymph melanization, and survival of larvae infected with (**c**) *C. albicans* or (**d**) *S. aureus* following anesthesia. Differences in immune parameters were analyzed by one-way ANOVA (**a**,**b**) or the Mantel–Cox logrank test (**c**,**d**) and were not statistically significant; n = 3 (**a**), n = 4 (**b**), n = 10 (**c**,**d**); data are shown as mean ± SEM (**a**,**b**) or as survival curves (**c**,**d**).

As inhalant anesthetics have a wide-ranging impact on vertebrate immune functions [22], given the frequent use of *G. mellonella* in infection research, it was assessed whether isoflurane would interfere with larval immune functions. At the optimal dose (2 μL/cm^3^), with immediate or delayed (5 min) removal from the chamber, no significant differences in hemocyte (immune cell) counts were observed compared to non-anesthetized controls, although a slight, not statistically significant decrease was noted with prolonged exposure (Figure 3a). Melanization, a key insect immune response to pathogens, combining both the humoral and cellular mechanisms, also remained unaffected (Figure 3b). When the survival of larvae infected with the fungi *C. albicans* or the bacterium *S. aureus* was tested, no differences were observed, apart from a slight but not statistically significant increase in *S. aureus*-induced mortality in the group with prolonged isoflurane exposure (Figure 3c,d).

## 4. Discussion

Isoflurane has already been tried for other insect anesthesia [12,14,15,16,17,23], due to its low cost and easy availability. Most investigators used it either at a 5% concentration, or at saturating concentrations (theoretically 31%, but practically closer to 20%). As precise dosing of anesthetic gas requires specialized apparatus, not always readily available in every laboratory, the current study relied on the use of an easily achievable saturating concentration, obtained by dosing 2 µL of isoflurane to evaporate per 1 cm^3^ of a tightly closed chamber. As quick anesthesia is the goal, and as there was a clear dose-dependent response in the time required for anesthesia of *G. mellonella* larvae, this saturating concentration of 2 μL/cm^3^ isoflurane was chosen for all subsequent experiments.

In *G. mellonella*, isoflurane appeared particularly effective, in comparison with other reported species. Previous attempts to use isoflurane for the anesthesia of large insects (Madagascar and giant cockroaches, thorny devil stick insects), either at a low 5% concentration, or at saturating concentrations, showed that over 30 min of exposure is needed to induce anesthesia [15,17]. A shorter time was needed for somewhat smaller budwing mantises [14]. In the case of *G. mellonella* larvae, it was even quicker: only one minute was needed for them to lose consciousness. This is similar to the previously reported quick isoflurane anesthesia of even smaller *Drosophila* flies [23], which suggests that there is a relationship between body size and speed of anesthesia onset. However, some possible peculiarities of *G. mellonella* or of larval anatomy and physiology (all other reports used adult insects) cannot be completely excluded as the reason for their quick response. Whatever the reason, this rapid onset minimizes the risk of distress prior to complete anesthesia, observed in larger insects [12,15].

This method is well-suited for simultaneous handling of multiple individuals or whole animal batches: anesthesia can be achieved even when the chamber is half-filled with larvae. For practical reasons, it is important for the anesthesia to last for a couple of minutes, which is needed to perform potentially painful procedures. Both in the case of *G. mellonella* larvae and in previously reported Drosophila [23], there was a correlation between the time spent in anesthetic gas after loss of consciousness and the time needed for the animals to recover from anesthesia. This is perhaps due to isoflurane gradually accumulating in larval fat tissues, which act as a depot-like source of anesthetic even after removal from the chamber, but more studies into such potential mechanisms are needed. Whatever the mechanism, extending the post-induction exposure to isoflurane by five minutes allows for anesthesia (from 5 min to over an hour, with 30 min being the average)—sufficient time to conduct painful terminal procedures (hemolymph collection, dissection).

In real experiments, where anesthesia will be applied to all experimental groups, any slight physiological effects induced by it should not interfere with interpretation of the results. Nevertheless, as *G. mellonella* larvae are so commonly used for infection experiments, it is worth knowing if isoflurane has any dramatic effect on larvae’s immune functions. Luckily, no such major effect of isoflurane anesthesia was observed in this study, neither with respect to the number of immune cells, the functional in vitro immune response such as melanization, nor the overall in vivo susceptibility to dying from an infection. This of course does not exclude some more nuanced effects—for example, in case of *Drosophila*, isoflurane was found to have a temporary effect on the stress resistance of anesthetized animals [23]. Therefore, it is probably always prudent to avoid planning experiments where any functions would be compared between animals that have and have not been anesthetized.

For the euthanasia of whole batches of animals (e.g., at the end of experiments), individual lethal injections, recommended for larger insects [13,15,17], are impractical; therefore, another euthanasia protocol is needed. Although isoflurane alone was insufficient for reliable *G. mellonella* euthanasia, consistent with reports in other insects [15,16,17], a two-step method proved effective, humane, and unlaborious: induction of anesthesia with isoflurane, and subsequent placing of the tube with unconscious larvae and residual anesthetic at −20 °C overnight. This is in line with recommendations from various animal welfare organizations, recommending two-step euthanasia protocols consisting of quick and effective anesthesia followed by a reliable lethal step [10,11].

In conclusion, the use of 2 μL/cm^3^ isoflurane, with an optional five-minute extension post-induction, offers an optimal method for the anesthesia of *G. mellonella* larvae, while the two-step protocol combining isoflurane anesthesia and subsequent freezing provides an efficient and ethical method for euthanasia.

## Data Availability

The original contributions presented in this study are included in the article. Further inquiries can be directed to the corresponding author.
